# Development of a prognostic model to predict BLCA based on anoikis-related gene signature: preliminary findings

**DOI:** 10.1186/s12894-023-01382-8

**Published:** 2023-12-04

**Authors:** Shusheng Zhu, Qingsong Zhao, Yanpeng Fan, Chao Tang

**Affiliations:** 1 Department of Urology, Jining No. 1 People’s Hospital, Jining, Shandong China; 2 Department of Urology, The First Hospital of Jilin University, Changchun, Jilin China; 3 Department of Urology, Affiliated Yantai Yuhuangding Hospital, Qingdao University, Yantai, 264000 Shandong China

**Keywords:** Bladder urothelial carcinoma, Anoikis, Gene, Risk model, Single-cell RNA sequencing

## Abstract

**Background:**

The prevalence of bladder urothelial carcinoma (BLCA) is significant on a global scale. Anoikis is a type of procedural cell death that has an important role in tumor invasion and metastasis. The advent of single-cell RNA sequencing (scRNA-seq) approaches has revolutionized the genomics field by providing unprecedented opportunities for elucidating cellular heterogeneity. Understanding the mechanisms associated with anoikis in BLCA is essential to improve its survival rate.

**Methods:**

Data on BLCA and clinical information were acquired from the databases of The Cancer Genome Atlas (TCGA) and Gene Expression Omnibus (GEO). ARGs were obtained from Genecards and Harmonizome databases. According to univariate Cox regression analysis, the least absolute shrinkage and selection operator (LASSO) algorithm was utilized to select the ARGs associated with the overall rate (OS). A multivariate Cox regression analysis was carried out to identify eight prognostic ARGs, leading to the establishment of a risk model. The OS rate of BLCA patients was evaluated using Kaplan–Meier survival analysis. To explore the molecular mechanism in low- and high-risk groups, we employed Gene Ontology (GO), Kyoto Encyclopedia of Genes and Genomes (KEGG), and Gene Set Enrichment Analysis (GSVA). Immune infiltration landscape estimation was performed using ESTIMATE, CIBERSOT, and single sample gene set enrichment analysis (ssGSEA) algorithms. Patients were categorized into different subgroups through consensus clustering analysis.

We employed biological functional enrichment analysis and conducted immune infiltration analysis to examine the disparities in potential biological functions, infiltration of immune cells, immune activities, and responses to immunotherapy.

**Results:**

We identified 647 ARGs and 37 survival-related genes. We further developed a risk scoring model to quantitatively assess the predictive capacity of ARGs. The high-risk score group exhibited an unfavorable prognosis, whereas the low-risk score group demonstrated a converse effect. We also found that the two groups of patients might respond differently to immune targets and anti-tumor drugs.

**Conclusion:**

The nomogram with 8 ARGs may help guide treatment of BLCA. The systematic assessment of risk scores can help to design more individualized and precise treatment strategies for BLCA patients.

**Supplementary Information:**

The online version contains supplementary material available at 10.1186/s12894-023-01382-8.

## Introduction

Bladder urothelial carcinoma (BLCA) ranks as the ninth most prevalent cancer globally, exhibiting a significant fatality rate. It is estimated that around 550,000 individuals are diagnosed with this condition annually [1, 2]. Patients with non-muscle invasive BLCA have a high rate of recurrence after surgery [3]. Muscle-invasive BLCA has a poor prognosis, with a 5-year overall survival rate of 40–50% [4]. Because there is a deficiency in identifiable biomarkers, a portion of individuals suffering from severe BLCA are still unable to receive effective treatment. Therefore, it is particularly important to construct an effective BLCA risk prediction model.

Recently, it has been discovered that during the process of metastasis, cancer cells transport extracellular matrix (ECM) while migrating and proliferating. Anoikis is triggered when tumor cells detach from the ECM [5]. Anoikis is a specific form of apoptosis, and its occurrence is an important mechanism of tumor invasion and metastasis [6]. However, cancer cells have the ability to develop various strategies to evade anoikis, including the utilization of growth factors, manipulation of pH levels, and adaptation to oxidative stress [7–9]. This discovery suggests ARGs may be exploited for cancer therapy.

Revealing novel avenues for addressing biological and medical challenges, single-cell RNA sequencing (scRNA-seq) presents unprecedented opportunities [10]. ScRNA-seq approaches have uncovered novel biology in terms of tissue composition, transcriptional dynamics, and regulatory relationships between genes [11]. Currently, scRNA-seq technology has become the most advanced method to reveal the heterogeneity and complexity of RNA transcripts within a single-cell [12]. Researchers can use scRNA-seq technology to build high-resolution profiles, which may provide better solutions for disease treatment.

In this study, we explored to analyze the relationship between the differential expression of ARGs and BLCA. Subsequently, a risk assessment model was developed to forecast the prognosis and immunotherapy outcomes for patients with BLCA. Finally, we performed scRNA-seq to build the foundation for the design of treatment regimens and the selection of targeted drugs. Exploring ARGs' prognostic model can help develop more personalized and accurate treatment strategies for BLCA patients. This study is entirely based on publicly available data.

## Materials and methods

### Data collection

Gene expression and clinical data for patients with BLCA were obtained from The Cancer Genome Atlas (TCGA) (https://portal.gdc.cancer.gov/) and Gene Expression Omnibus (GEO) (https://www.ncbi.nlm.nih.gov/ geo/query/acc.cgi?acc = GSE19423) databases. Limma is a software package in R/Bioconductor that offers a comprehensive solution for the analysis of gene expression experiment data [13]. The SVA package has the effect of identifying and removing batches [14]. The integration of the two sets of gene expression data was performed using the "limma" and "sva" packages.

### Identification of differential genes and characteristic differences of ARGs

ARGs were obtained from Genecards (https://www.genecards.org/) and Harmonizome (https://maayanlab.cloud/Harmonizome/) databases. Genecards and Harmonizome are public databases that can directly search for the required gene names. In the Genecards database, ARGs were selected with a relevance score > 0.4. All ARGs in the Harmonizome database were included. We utilized the limma package in R software to identify differentially expressed ARGs, using a threshold of |log2 fold change (FC)|> 1 and false discovery rate (FDR) < 0.05. The limma, survival and survminer packages were utilized for conducting survival analysis of AGRs. The RCircos package offers a convenient and versatile approach for generating R-based 2D track plots in the style of Circos [15]. CNV copy number data were obtained from UCSC Xena (https://xena.ucsc.edu/). We used RCircos package to further explore the alteration of these lost apoptosis-related genes on chromosomes and the location of each gene on chromosomes.

### ARGs subtype identification

In order to identify distinct patterns of ARGs, a clustering analysis was conducted using the "consensusClusterPlus" algorithm. According to the optimal classification of cluster number = 2, the patients with BLCA were clustered into two distinct molecular subtypes for further analysis. The principal component analysis (PCA) is a technique used to reduce the dimensionality of datasets, enhancing interpretability while minimizing information loss [16]. t-Distributed Stochastic Neighbor Embedding (tSNE) is a well-suited technique for the visualization of high-dimensional data [17]. The Uniform Manifold Approximation and Projection (UMAP) is a viable approach that can effectively decrease the dimensionality of beta diversity distance matrices [18]. PCA, tSNE and UMAP were plotted by the Rtsne, limma, ggplot2 and UMAP packages in R. The infiltration level of 23 types of immune cells was assessed using the "GSVA" R packages through a single sample gene set enrichment analysis (ssGSEA) algorithm. The "GSVA" R package was used to analyze the Gene Ontology (GO) and Kyoto Encyclopedia of Genes and Genomes (KEGG), enriching the ARGs in biological processes and signaling pathways. The distinct signal pathway between the two risk groups was described using gene set enrichment analysis (GSEA) to demonstrate the KEGG pathway enrichment.

### Construction and evaluation of risk score prognostic model

The training set was utilized to perform least absolute shrinkage and selection operator (LASSO) Cox regression analysis using the 'glmnet' package in R, aiming to develop a prognostic signature for ARG. Finally, we conducted multivariate Cox regression analysis to identify highly correlated genes and construct a prognostic gene signature. The risk score was calculated using the following formula: risk score = gene Cox coefficient (Coef) × gene expression (Exp). Where Coef was the regression coefficient and Exp was expression level of ARGs. We randomly divided samples into train group and test group in 1:1 ratio, classified the training group into high- and low-risk groups according to the median risk score [19, 20]. To assess the model's ability to predict outcomes, we utilized the "Survival ROC" R package to generate Kaplan–Meier (KM) survival curves and time-dependent receiver operating characteristic (ROC) curves.

The "rms" and "survival" R packages were utilized to construct a prognostic nomogram incorporating all independent prognostic factors. By summing the points assigned to each factor, the total points accurately predicted the 1-, 3-, and 5-year survival probabilities for patients with BLCA. Calibration curves and C-Index values were generated to assess the reliability of the survival prediction.

### Immune cell correlation analysis and drug sensitivity prediction

The ESTIMATE algorithm was utilized to calculate the stromal score, immune score, tumor purity, and ESTIMATE score in malignant tumor tissues for comparison between high-risk and low-risk groups [21]. The infiltration of immune cells and the presence of stroma in the tumor microenvironment (TME) were assessed using immune score and stromal score. The ESTIMATE score was calculated by summing up the stromal and immune scores to evaluate their combined effect. To determine the relative abundance of 22 different types of immune cells, cell-type identification by estimating relative subsets of RNA transcripts (CIBERSORT) analysis was employed [22]. The "oncoPredict" R package was utilized to compute the half-maximal inhibitory concentration (IC50) values of chemotherapeutic and targeted drugs for each BLCA sample.

### scRNA-seq and immunohistochemical (IHC) data extraction from public databases

The Tumor Immune Single-cell Hub 2 (TISCH2) is a scRNA-seq database (http://tisch.comp-genomics.org/) that aims to comprehensively characterize the TME at single-cell resolution [23]. To investigate the differences in ARG expression between normal and tumor tissues, we extracted the immunohistochemical results of 9 ARGs based on the public database of Human Protein Atlas (https://www.proteinatlas.org/). The flowchart of the present research is shown in Supplementary Figure S1.

### Statistical analysis

In our study, all statistical analysis was performed by R 4.1.3. Related R packages, including “ggplot2”, “limma”, “heatmap”, and “RColorBrewer”, and other related R packages were used. A *p* < 0.05 indicated statistically significant for all analyses (*p < 0.05; ** p < 0.01; ***p < 0.001).

## Results

### Identification of ARGs in BLCA

**Fig. 1:**
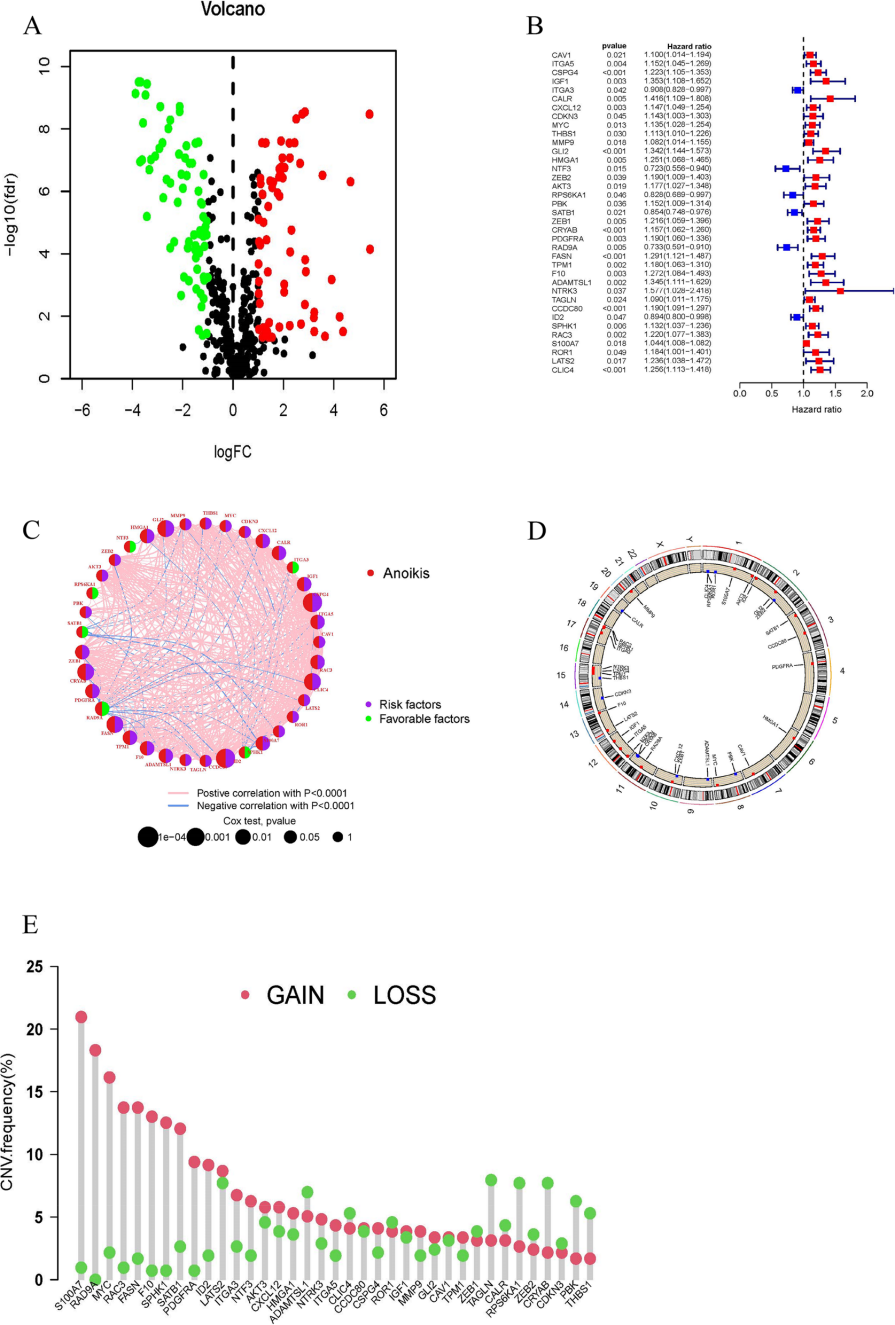
Genetic variations and expression of ARGs in BLCA. **A** Volcano plot of 137 DEGs, red for up-regulated, green for down-regulated genes. **B** The forest plot shows the 37 ARGs via the univariate Cox regression analysis. **C** Network diagram showing the interaction of 37 ARGs in BLCA. **D** The localization of the 37 ARGs on 23 chromosomes. **E** CNV variation frequency of 37 ARGs in BLCA

First, we obtained 647 ARGs from the public databases (Supplementary Table S1). The univariate analysis and survival analysis were conducted for ARGs. The volcano plot and forest plot were drawn (Fig. 1A, B). This study incorporated 37 ARGs and utilized network diagrams to depict the intricate correlation between varying ARGs and their prognostic significance in BLCA (Fig. 1C). The chromosomal visualization of CNV alterations in ARGs is depicted in Fig. 1D. In addition, CNV-associated mutations were prevalent among the 37 ARGs. S100A7, RAD9A, MYC, RAC3, FASN, F10, SPHK1, SATB1, PDGFRA and ID2 showed significant CNV amplification, but ADAMTSL1, TAGLN, RPS6KA1, CRYAB, PBK and THBS1 exhibited notable CNV deletions (Fig. 1E).

### Characteristics of different ARGs

**Fig. 2:**
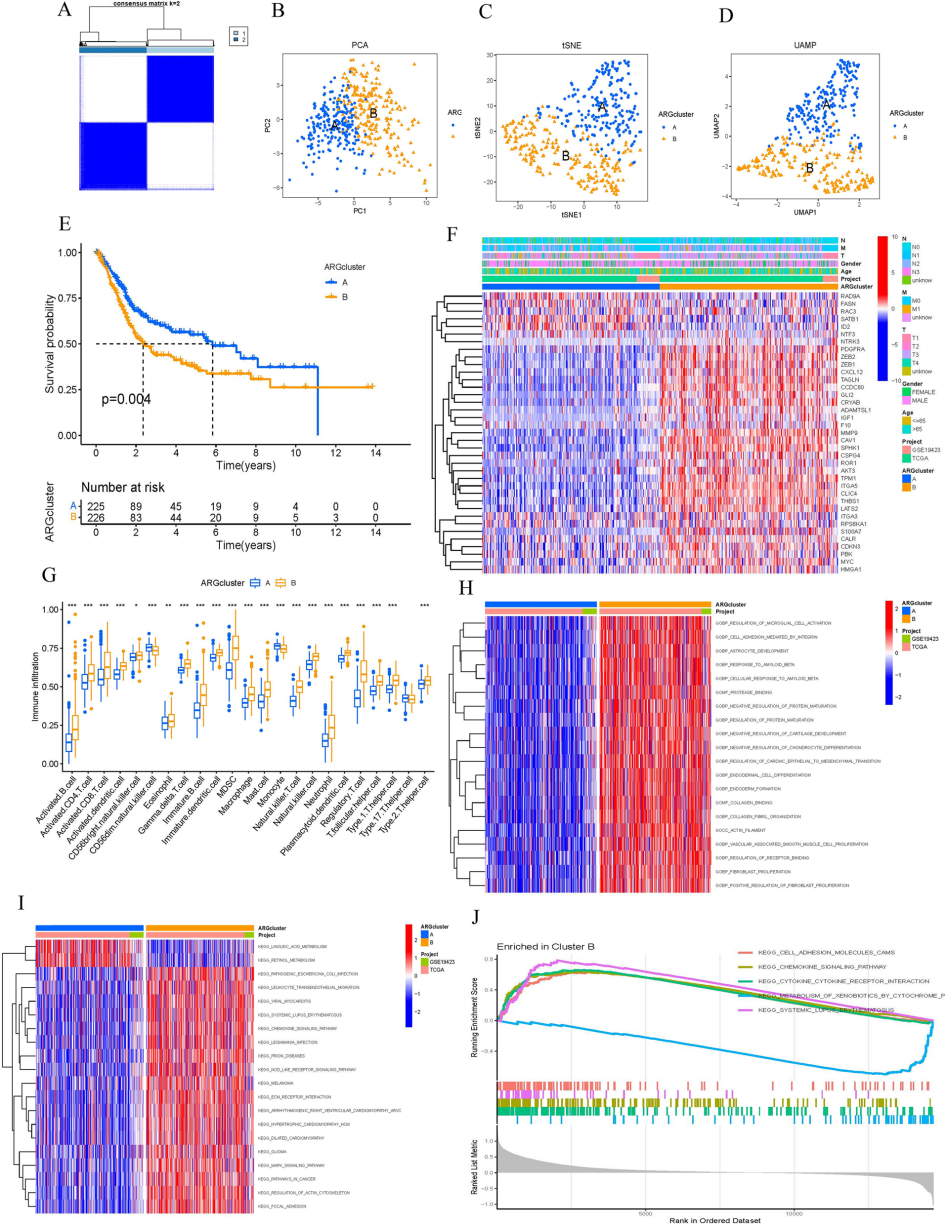
ARGs subtype identification. **A** Two ARG subtypes in CRC were identified by consensus clustering analysis. **B**-**D** PCA plot of risk score in ARGs cohort. **E** Survival analyses for the A and B groups. **F**-**G** Difference expression analysis and immune infiltration of ARGs in two groups. **H**-**J** GO and KEGG analyses for ARGs of the two groups

We categorized the BLCA patients into two distinct groups, labeled A and B, utilizing the NMF algorithm (Fig. 2A). The use of “PCA”, “tSNE”, and “UMAP” R packages could clearly distinguish between groups A and B (Fig. 2B-D). The Kaplan–Meier (KM) analysis revealed a significant disparity in patient survival rates between groups A and B (Fig. 2E). We also plotted a heatmap to visualize the detailed expression of ARGs in A and B groups (Fig. 2F). Regarding the differences in immune infiltration between the two groups, we observed a significant enrichment of the A group in Monocyte and CD56 cells (Fig. 2G). GO biopathway analysis showed that ARGs in the B group were obviously enriched in the following functional sets: regulation of microglia cell activation, cell adhesion mediated by integrin, astrocyte development and others (Fig. 2H). According to the KEGG analysis, B group exhibited significant activation of pathways related to cell adhesion molecules, chemokine signaling, cytokine-receptor interaction and systemic lupus erythematosus (Fig. 2I, J).

### The nomogram based on risk score in BLCA

**Fig. 3:**
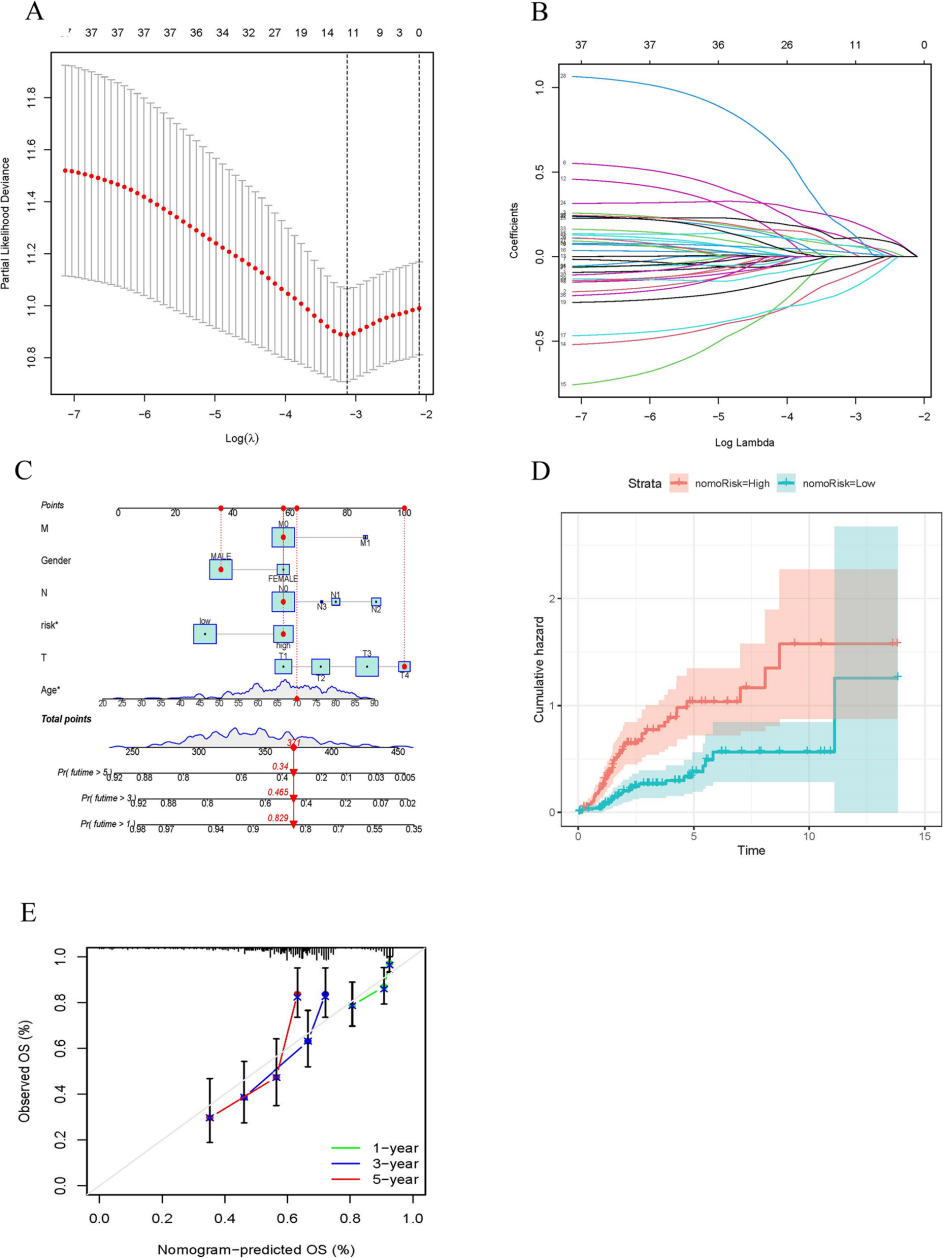
Construction of risk score prognostic model. **A**, **B** The least absolute shrinkage and selection operator (LASSO) method of ARGs associated with prognosis. **C** Nomogram construction based on the ARGs prognostic signature and clinicopathological characteristics. **D**, **E** Cumulative frequency and calibration curves for risk score model in the high and low group

To avoid overfitting, eight ARGs were further identified by the LASSO regression method (Fig. 3A, B). We constructed a risk score prognostic model by using 8 ARGs. Risk score = EXP _CSPG4_ × (0.164) + EXP _CALR_ × (-0.321) + EXP _NTF3_ × (-0.424) + EXP _RPS6KA1_ × (-0.446) + EXP _FASN_ × (0.316) + EXP _F10_ × (0.288) + EXP _NTRK3_ × (0.592) + EXP _S100A7_ × (0.073). Patients with BLCA were divided into low-risk and high-risk groups based on the median risk score. Finally, we built a nomogram to predicting the survival probabilities of BLCA (Fig. 3C-E).

### Evaluation of the risk score model

**Fig. 4:**
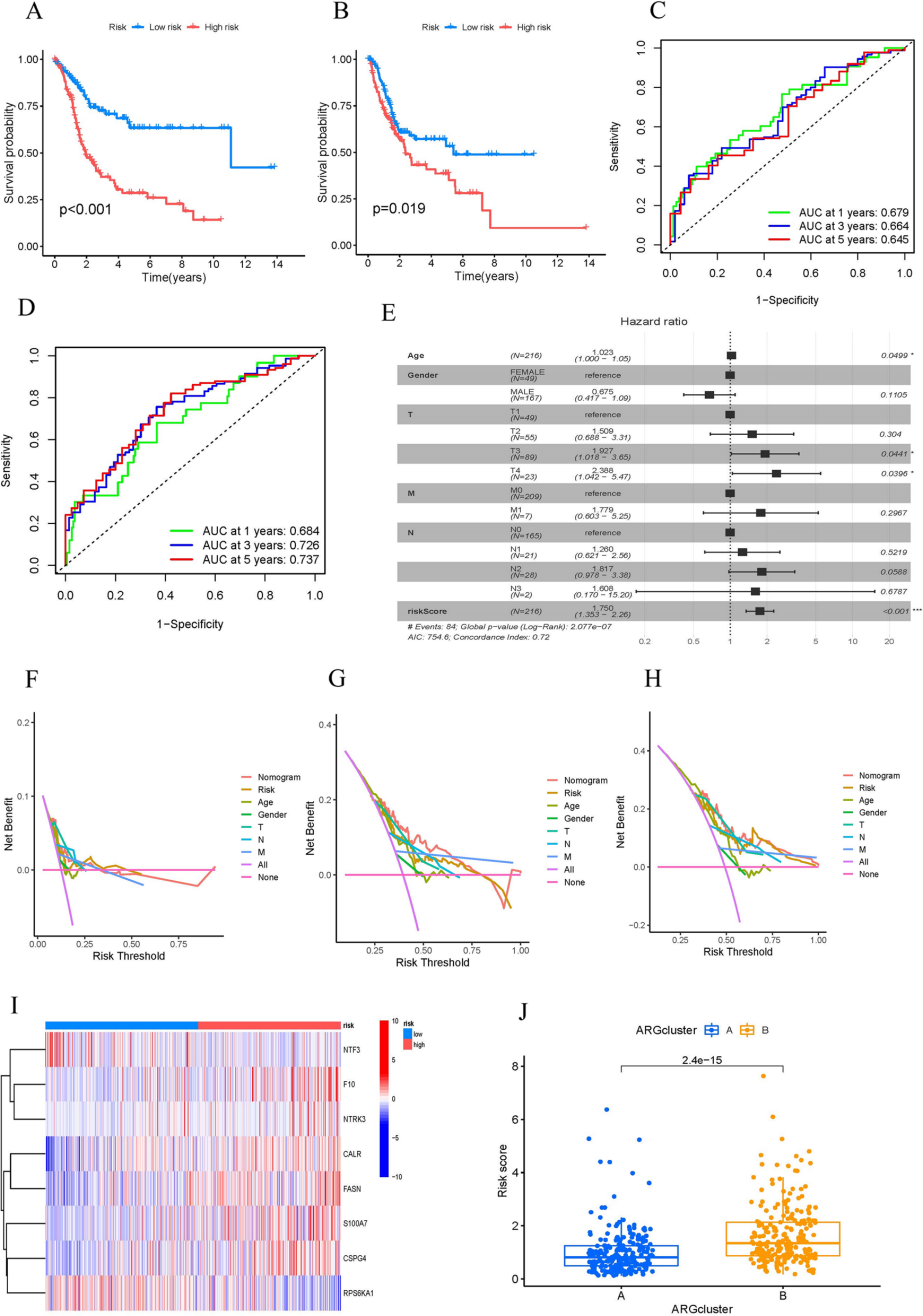
Validation of the risk score model. **A**, **B** Survival curve of the train and the test group. **C, D** Time-dependent receiver operating characteristics (ROC) of the train and the test group. **E** Forest plot of independent prognostic analysis. **F**–**H** The decision curve analysis. **I** Heatmap of ARGs expression between two groups. **J** Risk score of the two groups

Relevant findings from survival analysis and ROC curves indicated a less favorable prognosis in the high-risk group when compared to the low-risk group (Fig. 4A-D). To assess the validity of the risk prognostic model, we performed an independent prognostic analysis (Fig. 4E). The risk factor DCAs are presented in Fig. 4F-H. The heatmap revealed a higher level of ARG expression in the high-risk group as compared to the low-risk group (Fig. 4I). Moreover, we also found that group B had a higher risk score (Fig. 4J).

### Correlation of risk scores with immunotherapy response

**Fig. 5:**
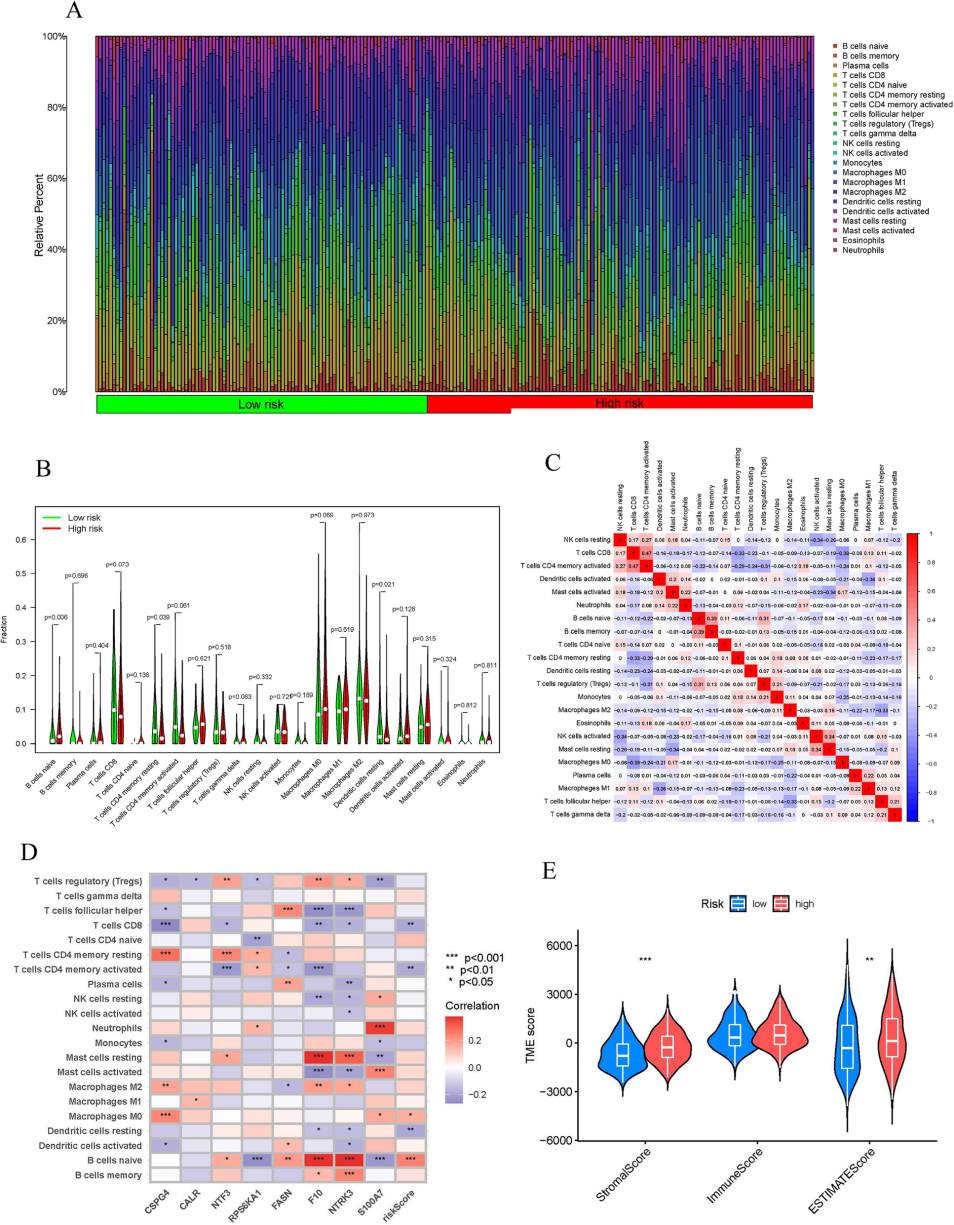
Correlation of risk scores with immunotherapy response. **A**, **B** Heatmap and vioplot based on two groups. **C** The correlation of risk score and immune infiltration cells. **D** Immune-related heatmap. **E** Vioplot of TME difference analysis

Immune cell differential analysis suggested that immune cell infiltration was different between the two groups, such as B cells naive, T cells CD4 memory resting and Dendritic cells resting (Fig. 5A, B). The correlation heatmap of immune cells was shown in Fig. 5C. We also assessed the value of risk scores in predicting response to immunotherapy. The correlation between risk scores and B cells naive and Macrophages M0 was found to be positive, while a negative correlation was observed with Dendritic cells resting, T cells CD4 memory activated, and T cells CD8 (Fig. 5D). TME difference analysis suggested significant differences in TME scores between the two groups in StromalScore and ESTIMATEScore (Fig. 5E).

### Drug sensitivity analysis

**Fig. 6:**
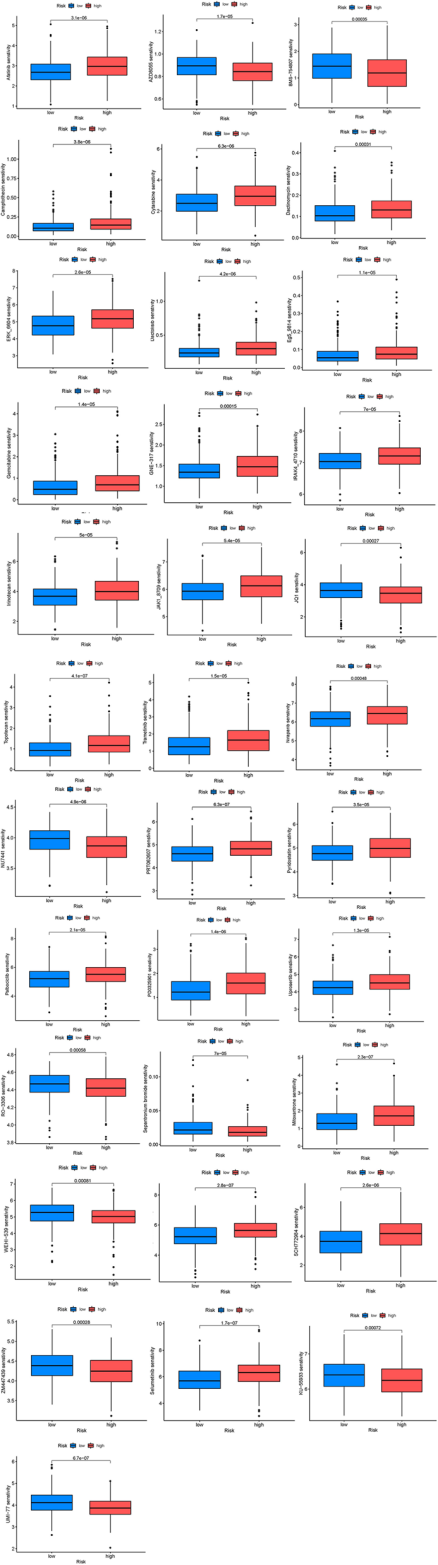
The relationship between drug risk scores and drug sensitivity

To search for the possibility of ARGs as prognostic markers in the individualized treatment of BLCA, we evaluated the relationship between drug risk scores and drug sensitivity in the treatment of BLCA. As shown in Fig. 6, the sensitivity of 34 anti-tumor drugs was significantly different in two groups (*p* < 0.05). This implies a potential role for these drugs in the future treatment of BLCA.

### ScRNA-seq and immunohistochemical (IHC) data extraction from public databases

**Fig. 7:**
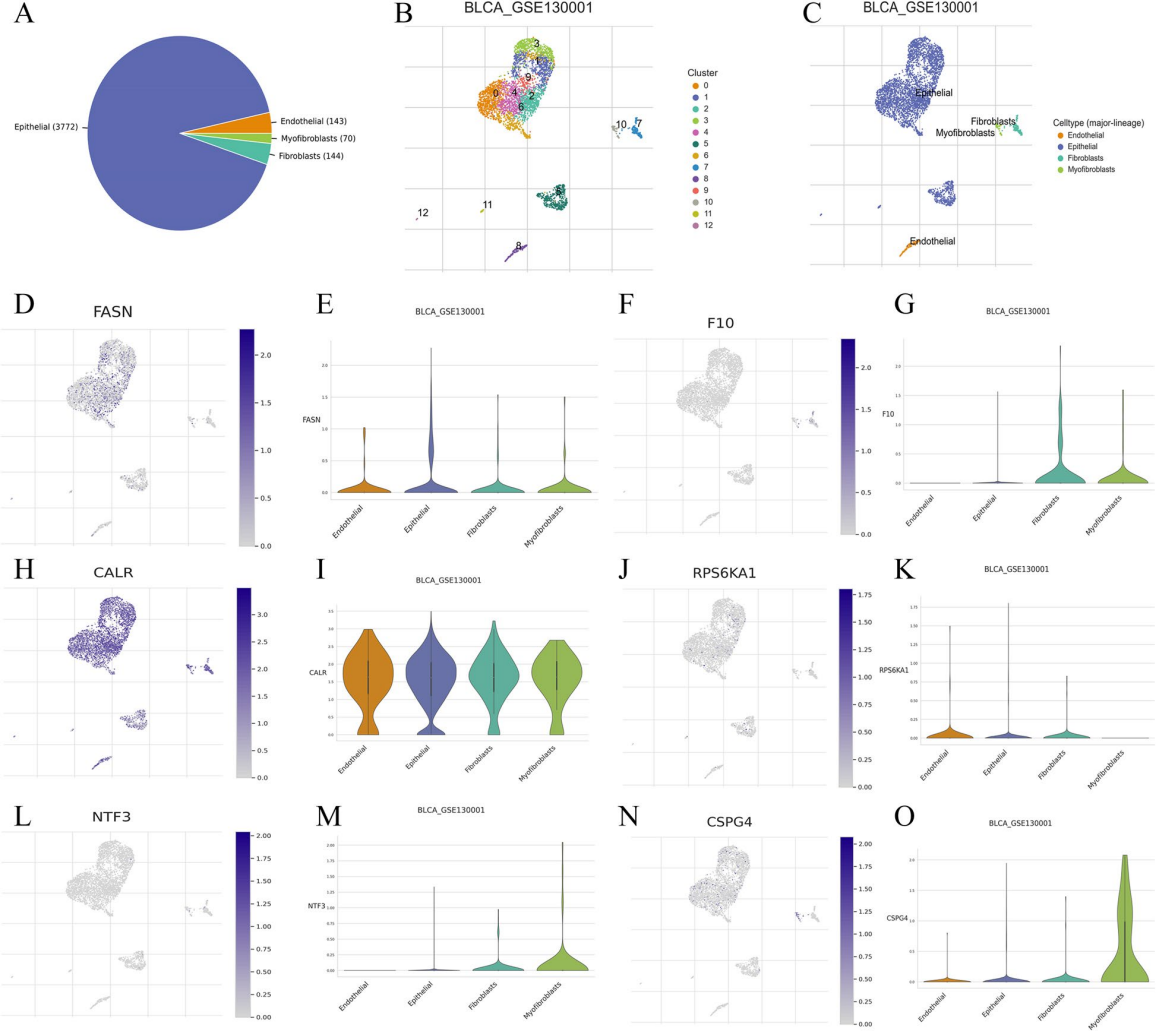
Single cell RNA sequencing (scRNA-seq) data extraction based on Tumor Immune Single-cell Hub 2 (TISCH2) database (http://tisch.comp-genomics.org/). **A**-**C** Basic data of BLCA_GSE130001. **D**-**O** ScRNA-seq data of FASN, F10, CALR, RPS6KA1, NTF3, CSPG4

**Fig. 8:**
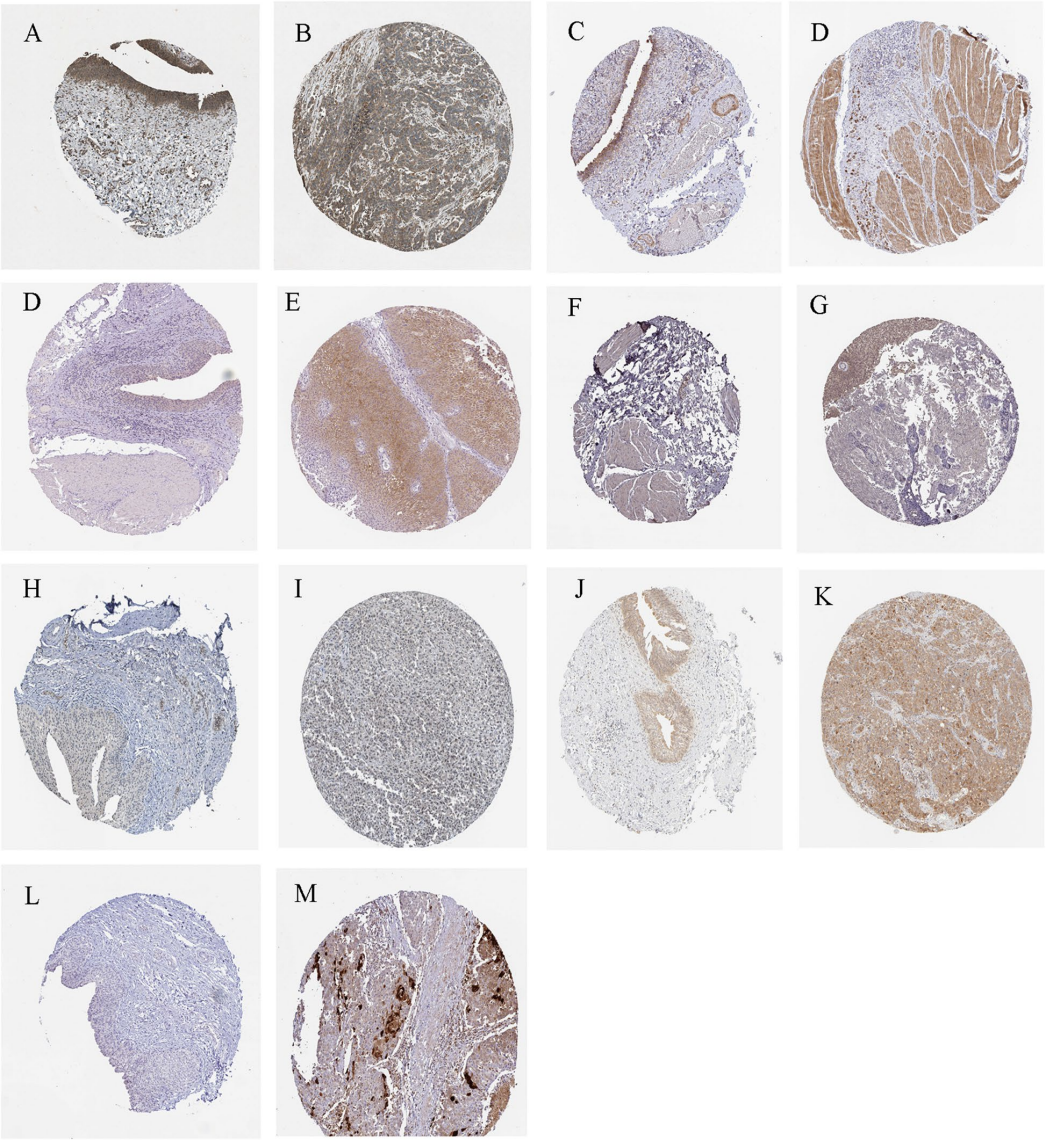
Immunohistochemical (IHC) data extraction based on public database (https:// www. proteinatlas.org/). Representative immunohistochemical staining for CALR, CSPG4, FASN, NTF3, NTRK3, RPS6KA1 and S100A7 in normal tissue and BLCA tissue

We obtained the gene expression data for two single-cell RNA sequencing samples from the GEO database using accession number GSE130001 (Fig. 7A-C). FASN was widely available in endothelial, epithelial, fibroblasts and myofibroblasts (Fig. 7 D, E). F10 was widely available in fibroblasts and myofibroblasts (Fig. 7 F, G). CALR was significantly expressed in endothelial, epithelial, fibroblasts and myofibroblasts (Fig. 7 H, I). RPS6KA1 was widely available in endothelial, epithelial and fibroblasts (Fig. 7 J, K). NTF3 was widely available in fibroblasts and myofibroblasts, minimally expressed in epithelial (Fig. 7 L, M). CSPG4 was widely available in myofibroblasts, minimally expressed in endothelial, epithelial and fibroblasts (Fig. 7 N, O). IHC data revealed a notable upregulation of ARG expression levels in BLCA tissues compared to normal tissues (Fig. 8).

## Discussion

Anoikis, a programmed cell death caused by cell detachment from the extracellular matrix, is an important mechanism that prevents cell growth and attachment to inappropriate substrates independent of adhesions, thereby avoiding colonization of distant organs [24–26]. The scientific community has shown significant interest in the capacity of tumor cells to evade anoikis, given its crucial role in tumor progression and metastatic establishment [24, 27]. Intracellular signals such as cellular DNA damage and stress in the endoplasmic reticulum have the potential to induce anoikis, with mitochondria playing a pivotal role in regulating apoptosis [28]. This malfunction in the implementation of anoikis could potentially serve as an indicator for the invasion and movement of cancer cells, while also playing a role in the creation of metastases in distant organs and the emergence of resistance to drugs [29]. Hence, comprehending the mechanism underlying resistance to anoikis can aid in impeding tumor advancement and averting metastasis [6, 30].

In our research, we discovered 37 prognostic ARGs. Through LASSO analysis, out of which 8 were chosen as predictive models for estimating the overall survival (OS) in patients with bladder cancer (BLCA). The BLCA patients were classified using the consensus clustering algorithm, resulting in the identification of two potential subgroups. We conducted a comparative analysis of the data from both groups, revealing contrasting survival durations among BLCA patients in each group. Furthermore, there were notable disparities observed in terms of immune cell infiltration and immune targets among the two cohorts. Next, we constructed risk models based on 8 ARGs as a way to predict the survival and prognosis of BLCA patients. Based on the outcomes of ROC curves and an independent prognostic analysis, our constructed prognostic model demonstrated excellent predictive accuracy.

Growing evidence showed that the existence of anti-ARGs was strongly associated with tumor aggressiveness and tumor drug resistance [9, 31–33]. A study indicated that lncRNA APOC1P1-3 could promote breast cancer metastasis by specifically binding miRNA-188-3p to block Bcl-2 inhibition through anoikis-resistance [34]. After stromal deprivation, the lymphocyte spectrum specific Ets transcription factor SPIB was activated and directly enhanced SNAP47 transcription in certain lung cancer cells. Loss of adhesion-induced autophagy significantly increased anoikis-resistance through SPIB activation [26]. Here, we found ARGs alterations at the transcriptional level and showed a reciprocal correlation of BLCA. Down-regulation of NTF3 and RPS6KA1 genes had a positive effect on the survival of BLCA patients, and up-regulation of the other 6 genes had a negative effect on the survival of BLCA patients. Egan et al. found significantly elevated CSPG4 expression in aggressive thyroid cancer, which was strongly associated with poor prognosis [35]. CALR mutations had an important role in myeloproliferative neoplasms [36, 37]. NTF3 also had a significant correlation with prognosis and immune infiltration in hepatocellular carcinoma [38]. A research study demonstrated that the activation of the PI3K-Akt-mTOR pathway was facilitated by the interaction between the FLT3-ITD mutation and the CXCL12/CXCR4 axis. This activation, in turn, resulted in an increase in RPS6KA1 expression which regulated the expression of genes associated with resistance to multiple drugs, thereby contributing to drug indications [39]. Magali et al. suggested that FASN could be used for the de novo synthesis of fatty acids in human adipogenic enzymes, which were highly expressed in cancer cells [40]. In glioma patients, hypomethylation of the F10 promoter was responsible for the overexpression and aggressive biological behavior of the F10-encoded protein FX [41]. The NTRK3 gene was involved in regulating the malignant behavior of malignant melanoma and might be a new therapeutic target for malignant melanoma [42]. S100A7 could promote esophageal squamous cell carcinoma progression by activating oncogenic pathways and remodeling the TME [43]. Therefore, exploring ARGs is more useful for developing more personalized immunotherapy programs.

Finally, we performed a differential analysis of TME and drug sensitivity based on risk score prognostic model. We observed that BLCA patients with lower risk scores had significantly longer OS. When studying treatment outcomes based on risk scores in BLCA patients, we found correlation between drug sensitivity and risk score. This discovery validates the dependability of employing this risk score model for prognosticating the effectiveness of immunotherapy in patients with BLCA. In general, as a new prognostic risk model, the studies on these eight ARGs are superficial and further research is necessary in the future. To further validate the risk model, general patient information, including age, sex, tumor stage, and score of sensitive genes, should be collected. Our single-cell sequencing data indicated that ARGs were expressed to varying degrees in different cells of BLCA patients. Furthermore, we offer a comprehensive analysis of the contrasting expression patterns of BLCA marker genes in both tumor and normal tissue. IHC showed that the expression level of ARGs in the BLCA tissue was significantly higher than that in the normal tissue. However, there are few reports on F10. Therefore, it is necessary to further investigate the ARGs F10 in future research.

Needless to say, the study still has some limitations. First, the investigation exclusively utilized openly accessible datasets, potentially introducing selection bias. Secondly, all the findings in this manuscript were of speculation based on the transcriptional analyses using bioinformatics analysis, the exact mechanism of ARGs in BLCA need to be further investigated in vivo and in vitro. Additionally, there is a deficiency of crucial experimental evidence to authenticate the manifestation of predictive genes in BLCA. Thus, it is necessary to further explore the specific mechanism of action of ARGs through molecular and animal experiments.

## Conclusion

In this study, we systematically investigated the prognostic model of BLCA risk score based on 8 ARGs and linked these models to TME, drug sensitivity, scRNA-seq, and IHC. The prognostic model based on risk scoring demonstrated its efficacy in accurately forecasting the prognosis of patients with BLCA and their response to immunotherapy. At the same time, the systematic assessment of risk scores could help to design more individualized and precise treatment strategies for BLCA patients.

### Supplementary Information

**Additional file 1: Supplementary Figure S1.** Flowchart of the present research.

**Additional file 2: Supplementary Table S1.** 647 anoikis-related genes.

## Data Availability

The study data are available from the TCGA database (https://portal.gdc.cancer.gov/) and the GEO (GSE19423) database (https://www.ncbi.nlm.nih.gov/geo/). ARGs were obtained from Genecards database (https://www.genecards.org/) and Harmonizome database (https://maayanlab.cloud/Harmonizome/). Single-cell sequencing based on Tumor Immune Single-cell Hub 2 database (http://tisch.comp-genomics.org/). IHC based on Human Protein Atlas (https://www.proteinatlas.org/). The anoikis-related genes can be found in the Supplementary Material. Further inquiries can be directed to the corresponding author.

## Abbreviations

BLCA: Bladder urothelial carcinoma
scRNA-seq: Single-cell RNA sequencing
ARGs: Anoikis-related genes
TCGA: The Cancer Genome Atlas
GEO: Gene Expression Omnibus
KEGG: Kyoto Encyclopedia of Genes and Genomes
GO: Gene ontology
PCA: Principal component analysis
IC50: Half-maximal inhibitory concentration prediction
ECM: Extracellular matrix
LASSO: Least absolute shrinkage and selection operator
TME: Tumor microenvironment
IHC: Immunohistochemical
