# Supplementary figures and images for: Development of a prognostic model to predict BLCA based on anoikis-related gene signature: preliminary findings
Source: BMC Urol. 2023 Dec 4;23:199. doi: 10.1186/s12894-023-01382-8 (PMC10694890; doi:10.1186/s12894-023-01382-8)

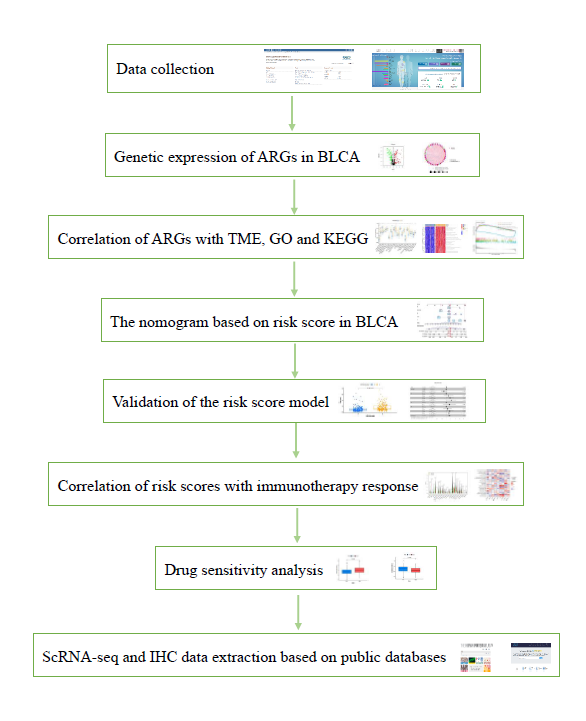


Supplementary Figure S1: Flowchart of the present research.

Supplement: Supplementary file 1 — Additional file 1: Supplementary Figure S1. Flowchart of the present research. [file 12894_2023_1382_MOESM1_ESM.docx]
