# Supplementary material for: Development of a prognostic model to predict BLCA based on anoikis-related gene signature: preliminary findings
Source: BMC Urol. 2023 Dec 4;23:199. doi: 10.1186/s12894-023-01382-8 (PMC10694890; doi:10.1186/s12894-023-01382-8)
Supplement: Supplementary file 2 — Additional file 2: Supplementary Table S1. 647 anoikis-related genes. [file 12894_2023_1382_MOESM2_ESM.docx]

Supplementary Table S1: 647 anoikis-related genes.

**Genes**

| 1. BRMS1 2. PTK2 |
| --- |
| 1. NTRK2 |
| 1. BCL2L11 |
| 1. SRC |
| 1. CEACAM6 |
| 1. CAV1 |
| 1. AKT1 |
| 1. ITGB1 |
| 1. CEACAM5 |
| 1. EGFR |
| 1. BCL2 |
| 1. CASP8 |
| 1. SIK1 |
| 1. PTRH2 |
| 1. STAT3 |
| 1. TLE1 |
| 1. CTNNB1 |
| 1. DAPK2 |
| 1. ZNF304 |
| 1. MAPK1 |
| 1. BMF |
| 1. ITGA5 |
| 1. TP53 |
| 1. MCL1 |
| 1. CASP3 |
| 1. CDH1 |
| 1. BAD |
| 1. MAPK3 |
| 1. PAK1 |
| 1. PIK3CA |
| 1. ITGAV |
| 1. FN1 |
| 1. PTGS2 |
| 1. BCL2L1 |
| 1. BAX |
| 1. BCAR1 |
| 1. ERBB2 |
| 1. PTEN |
| 1. ANGPTL4 |
| 1. PDK4 |
| 1. CYCS |
| 1. BRAF |
| 1. YAP1 |
| 1. ANKRD13C |
| 1. ITGA2 |
| 1. ANXA5 |
| 1. BIRC5 |
| 1. TIMP1 |
| 1. MTOR |
| 1. ATF4 |
| 1. BDNF |
| 1. CSPG4 |
| 1. BSG |
| 1. AKT2 |
| 1. MAPK8 |
| 1. IGF1 |
| 1. IGF1R |
| 1. STK11 |
| 1. ITGA6 |
| 1. ILK |
| 1. CFLAR |
| 1. RHOA |
| 1. HIF1A |
| 1. DAP3 |
| 1. MYBBP1A |
| 1. ITGA3 |
| 1. PTK2B |
| 1. TLE5 |
| 1. CCND1 |
| 1. CTTN |
| 1. CALR |
| 1. CDCP1 |
| 1. PLAUR |
| 1. SKP2 |
| 1. CHEK2 |
| 1. HGF |
| 1. EGF |
| 1. E2F1 |
| 1. PIK3CG |
| 1. ITGB4 |
| 1. DAPK1 |
| 1. PIK3R1 |
| 1. PIK3R3 |
| 1. MAP2K1 |
| 1. CXCL12 |
| 1. LGALS3 |
| 1. FBXW7-AS1 |
| 1. BAK1 |
| 1. ABHD4 |
| 1. CD44 |
| 1. ITGA4 |
| 1. FADD |
| 1. PHLDA2 |
| 1. TGFB1 |
| 1. HMCN1 |
| 1. MMP2 |
| 1. CEBPB |
| 1. CEMIP |
| 1. CDKN3 |
| 1. CBL |
| 1. CASP9 |
| 1. SFN |
| 1. MTDH |
| 1. PRKCA |
| 1. TNFRSF10B |
| 1. CXCL8 |
| 1. MIR200C |
| 1. AR |
| 1. CDKN2A |
| 1. CPT1A |
| 1. PIK3CB |
| 1. CLDN1 |
| 1. MIR204 |
| 1. MIR26A1 |
| 1. CDKN1A |
| 1. CDKN1B |
| 1. KLF12 |
| 1. NTRK1 |
| 1. PDGFRB |
| 1. MYC |
| 1. PLAU |
| 1. SMAD4 |
| 1. PLK1 |
| 1. MUC1 |
| 1. LGALS1 |
| 1. PYCARD |
| 1. SESN2 |
| 1. ITGB3 |
| 1. KRAS |
| 1. BID |
| 1. THBS1 |
| 1. HRAS |
| 1. CDK11B |
| 1. CDK11A |
| 1. XIAP |
| 1. PPARG |
| 1. IL6 |
| 1. MIR145 |
| 1. MYH9 |
| 1. CCR7 |
| 1. MSLN |
| 1. RAC1 |
| 1. GRHL2 |
| 1. BIRC3 |
| 1. RHOG |
| 1. CCAR2 |
| 1. NQO1 |
| 1. NOTCH1 |
| 1. MMP13 |
| 1. FAS |
| 1. MTA1 |
| 1. MYO5A |
| 1. EDA2R |
| 1. CCN6 |
| 1. MMP9 |
| 1. ABL1 |
| 1. MAPK11 |
| 1. SOD2 |
| 1. PTHLH |
| 1. PDGFB |
| 1. GLI2 |
| 1. EZH2 |
| 1. RIPK1 |
| 1. CXCR4 |
| 1. HMGA1 |
| 1. SIK2 |
| 1. TNFSF10 |
| 1. ANGPTL2 |
| 1. S100A4 |
| 1. NTF3 |
| 1. ETV4 |
| 1. MIR21 |
| 1. MIR124-1 |
| 1. HTRA1 |
| 1. LATS1 |
| 1. CEACAM3 |
| 1. EIF2AK3 |
| 1. LAMC2 |
| 1. LAMB3 |
| 1. LAMA3 |
| 1. CDH2 |
| 1. CSNK2A1 |
| 1. EDIL3 |
| 1. ZEB2 |
| 1. TLN1 |
| 1. EPHA2 |
| 1. SIRT3 |
| 1. OLFM3 |
| 1. CLU |
| 1. SPINK1 |
| 1. CPEB2 |
| 1. NAT1 |
| 1. TSG101 |
| 1. MIR200A |
| 1. MIR6744 |
| 1. SERPINA1 |
| 1. AKT3 |
| 1. RELA |
| 1. MAPK14 |
| 1. RPS6KA1 |
| 1. TNFRSF1A |
| 1. FASLG |
| 1. AFP |
| 1. ITGA8 |
| 1. PBK |
| 1. SATB1 |
| 1. CD63 |
| 1. EEF1A1 |
| 1. NOX4 |
| 1. LTB4R2 |
| 1. MAVS |
| 1. HRC |
| 1. CCN2 |
| 1. RHOB |
| 1. PPP1R13B |
| 1. MET |
| 1. PLG |
| 1. RAF1 |
| 1. PARP1 |
| 1. MAPK10 |
| 1. PRKCQ |
| 1. RB1 |
| 1. BRCA2 |
| 1. SP1 |
| 1. HAVCR2 |
| 1. VTN |
| 1. DOCK1 |
| 1. PDCD4 |
| 1. INHBB |
| 1. RANBP9 |
| 1. PRPF4B |
| 1. SESN1 |
| 1. SESN3 |
| 1. ZBTB7A |
| 1. CD24 |
| 1. MIR141 |
| 1. ELANE |
| 1. IDH1 |
| 1. KDR |
| 1. MDM2 |
| 1. NFE2L2 |
| 1. ZEB1 |
| 1. KL |
| 1. CRYAB |
| 1. PRKCI |
| 1. FGF2 |
| 1. HK2 |
| 1. LTF |
| 1. EPHB6 |
| 1. IQGAP1 |
| 1. MGAT5 |
| 1. SDCBP |
| 1. ABHD2 |
| 1. SPIB |
| 1. TRIM31 |
| 1. MIR1827 |
| 1. PDGFRA |
| 1. TLR3 |
| 1. NRAS |
| 1. PLAT |
| 1. ROCK1 |
| 1. VEGFA |
| 1. CASP10 |
| 1. PAK4 |
| 1. IL1RAP |
| 1. PIN1 |
| 1. TWIST1 |
| 1. UBE2C |
| 1. YWHAZ |
| 1. BMP6 |
| 1. ELK1 |
| 1. BNIP3L |
| 1. KDM3A |
| 1. BNIP3 |
| 1. PRDX4 |
| 1. LMO3 |
| 1. ZNF32 |
| 1. MIR200B |
| 1. MIR525 |
| 1. MIR363 |
| 1. TUBB3 |
| 1. HSP90B1 |
| 1. PTPN11 |
| 1. SLC2A1 |
| 1. HMOX1 |
| 1. PRKACA |
| 1. PAK3 |
| 1. CD36 |
| 1. PIK3R2 |
| 1. PPP2CA |
| 1. CASP6 |
| 1. PAK2 |
| 1. CDH3 |
| 1. PTK6 |
| 1. EEF2K |
| 1. LPAR1 |
| 1. LRP1 |
| 1. TCF7L2 |
| 1. TGFBR3 |
| 1. GLO1 |
| 1. RBL2 |
| 1. SIRPA |
| 1. CEACAM1 |
| 1. GDF2 |
| 1. TRAF2 |
| 1. ADCY10 |
| 1. IL17A |
| 1. VPS37A |
| 1. TNFRSF12A |
| 1. APOBEC3G |
| 1. BAG1 |
| 1. COL13A1 |
| 1. MNX1 |
| 1. RAD9A |
| 1. IFI27 |
| 1. MEGF11 |
| 1. ITPRIP |
| 1. BCL2L15 |
| 1. SNAI2 |
| 1. NOTCH3 |
| 1. PTPN1 |
| 1. GLUD1 |
| 1. SIRT1 |
| 1. FASN |
| 1. RPS6KB1 |
| 1. TPM1 |
| 1. PPP2R1A |
| 1. COL4A2 |
| 1. CTNND1 |
| 1. CD151 |
| 1. MMP11 |
| 1. SEMA7A |
| 1. PPP2R2A |
| 1. ARHGEF7 |
| 1. CCN1 |
| 1. BST2 |
| 1. PPP2R5A |
| 1. PPP2R2D |
| 1. CCDC178 |
| 1. MIR10A |
| 1. MIR30B |
| 1. MIR30C1 |
| 1. SHC1 |
| 1. BUB1 |
| 1. CDC25C |
| 1. CDK1 |
| 1. BUB3 |
| 1. ITGB5 |
| 1. SETD2 |
| 1. FER |
| 1. TP73 |
| 1. MAD2L1 |
| 1. SLCO1B3 |
| 1. DLG1 |
| 1. PDCD6IP |
| 1. BCL2L2 |
| 1. TDGF1 |
| 1. EDAR |
| 1. SH3GLB1 |
| 1. SCRIB |
| 1. DYNLL2 |
| 1. TSC2 |
| 1. BAG4 |
| 1. MAP3K7 |
| 1. F10 |
| 1. F3 |
| 1. ADAMTSL1 |
| 1. SERPINB1 |
| 1. MIR181A1 |
| 1. MAP3K1 |
| 1. CTBP1 |
| 1. CEACAM4 |
| 1. PRKD1 |
| 1. PXN |
| 1. MALAT1 |
| 1. GSTP1 |
| 1. PRDX1 |
| 1. SERPINE1 |
| 1. FOXO3 |
| 1. IKBKG |
| 1. TFDP1 |
| 1. CRYBA1 |
| 1. ACTG1 |
| 1. ARHGDIA |
| 1. EZR |
| 1. SLC39A6 |
| 1. BIN1 |
| 1. TIAM1 |
| 1. CRABP2 |
| 1. PDPK1 |
| 1. SMAD7 |
| 1. NTRK3 |
| 1. RHOC |
| 1. CASP2 |
| 1. TNC |
| 1. IRF6 |
| 1. HOTAIR |
| 1. GNE |
| 1. XAF1 |
| 1. SFRP1 |
| 1. MAP2K2 |
| 1. CSK |
| 1. PIK3C2B |
| 1. ENDOG |
| 1. RACK1 |
| 1. TAGLN |
| 1. FOXC2 |
| 1. ARHGDIB |
| 1. FBLIM1 |
| 1. CCDC80 |
| 1. LDHA |
| 1. ANXA2 |
| 1. BLNK |
| 1. SPP1 |
| 1. SMARCE1 |
| 1. QSOX1 |
| 1. RBFOX2 |
| 1. RPS6KA3 |
| 1. CDC42 |
| 1. JUP |
| 1. MAOA |
| 1. PIP5K1C |
| 1. ATF2 |
| 1. NKX2-1 |
| 1. NDRG1 |
| 1. OCLN |
| 1. ID2 |
| 1. CEACAM8 |
| 1. AFAP1L1 |
| 1. PITPNC1 |
| 1. INSR |
| 1. HSPB1 |
| 1. NGF |
| 1. GSK3B |
| 1. PCNA |
| 1. KRT14 |
| 1. SPHK1 |
| 1. TP63 |
| 1. CTNNA1 |
| 1. EHMT2 |
| 1. SIRT6 |
| 1. FOXA1 |
| 1. OGT |
| 1. RAC3 |
| 1. ACP1 |
| 1. STK38 |
| 1. RHOQ |
| 1. MUC4 |
| 1. SRSF3 |
| 1. ONECUT1 |
| 1. S100A7 |
| 1. GKN1 |
| 1. MIR107 |
| 1. MIR630 |
| 1. DNMT1 |
| 1. LCK |
| 1. MERTK |
| 1. UCHL1 |
| 1. CDK2 |
| 1. MMP3 |
| 1. ACTB |
| 1. BRCA1 |
| 1. NOS2 |
| 1. SLC2A2 |
| 1. USP9X |
| 1. FYN |
| 1. HSPA1A |
| 1. ROR1 |
| 1. HTRA2 |
| 1. SKI |
| 1. C5AR1 |
| 1. LATS2 |
| 1. PRDM1 |
| 1. SNAI1 |
| 1. SPTA1 |
| 1. TJP1 |
| 1. TPP2 |
| 1. XRCC5 |
| 1. CLDN18 |
| 1. THY1 |
| 1. DOK2 |
| 1. SERPINB5 |
| 1. CDX2 |
| 1. CENPF |
| 1. IKZF3 |
| 1. S100A11 |
| 1. SNCG |
| 1. SRPX2 |
| 1. USP11 |
| 1. ELAVL1 |
| 1. HOXA10 |
| 1. LGALS8 |
| 1. SLPI |
| 1. CLIC4 |
| 1. HTRA3 |
| 1. EFHD2 |
| 1. IRX1 |
| 1. CXCL14 |
| 1. KIF18A |
| 1. ZG16B |
| 1. SBSN |
| 1. MIR223 |
| 1. MIR99A |
| 1. MIR503 |
| 1. MIR451A |
| 1. MIR7-1 |
| 1. SNORA80E |
| 1. BMF |
| 1. DAPK2 |
| 1. E2F1 |
| 1. STK11 |
| 1. TFDP1 |
| 1. ABHD4 |
| 1. AFAP1L1 |
| 1. AKT1 |
| 1. AKT2 |
| 1. ANGPTL4 |
| 1. BAX |
| 1. BCAR1 |
| 1. BCL2 |
| 1. BCL2L11 |
| 1. BIRC3 |
| 1. BMF |
| 1. BRAF |
| 1. BRCA2 |
| 1. BSG |
| 1. CALR |
| 1. CASP2 |
| 1. CASP3 |
| 1. CASP8 |
| 1. CAV1 |
| 1. CCAR2 |
| 1. CD63 |
| 1. CDCP1 |
| 1. CDH1 |
| 1. CDH2 |
| 1. CDKN2A |
| 1. CEACAM6 |
| 1. CEBPB |
| 1. CHUK |
| 1. CLDN1 |
| 1. CLU |
| 1. CMA1 |
| 1. COPS5 |
| 1. CSNK2A1 |
| 1. CSPG4 |
| 1. CTNND1 |
| 1. CTTN |
| 1. CXCL12 |
| 1. DAP3 |
| 1. DAPK1 |
| 1. DLG1 |
| 1. EDA2R |
| 1. EEF1A1 |
| 1. EEF2K |
| 1. EGFR |
| 1. EIF2AK3 |
| 1. ERBB4 |
| 1. FER |
| 1. FGF2 |
| 1. FN1 |
| 1. HGF |
| 1. HK2 |
| 1. HMCN1 |
| 1. HMGA1 |
| 1. HOXA10 |
| 1. HTRA1 |
| 1. IGF1R |
| 1. IKZF3 |
| 1. ITGA2 |
| 1. ITGA3 |
| 1. ITGA4 |
| 1. ITGA5 |
| 1. ITGA6 |
| 1. ITGA8 |
| 1. ITGAV |
| 1. ITGB1 |
| 1. KDR |
| 1. KL |
| 1. KRAS |
| 1. LGALS1 |
| 1. LRP1 |
| 1. LTB4R2 |
| 1. MAPK1 |
| 1. MAPK3 |
| 1. MAVS |
| 1. MCL1 |
| 1. MDM2 |
| 1. MET |
| 1. MGAT5 |
| 1. MIR200C |
| 1. MMP11 |
| 1. MMP13 |
| 1. MMP2 |
| 1. MTA1 |
| 1. MTOR |
| 1. MYBBP1A |
| 1. NRP1 |
| 1. NTF3 |
| 1. NTRK2 |
| 1. OLFM3 |
| 1. PAK1 |
| 1. PAK4 |
| 1. PECAM1 |
| 1. PIK3CA |
| 1. PIK3CG |
| 1. PLK1 |
| 1. PRKCA |
| 1. PRKD1 |
| 1. PTEN |
| 1. PTHLH |
| 1. PTK2 |
| 1. PTK2B |
| 1. PTK6 |
| 1. PTPN11 |
| 1. PTRH2 |
| 1. RAD9A |
| 1. RHOA |
| 1. RHOC |
| 1. RIPK1 |
| 1. ROCK1 |
| 1. S100A4 |
| 1. SCRIB |
| 1. SH3GLB1 |
| 1. SIK1 |
| 1. SIRPA |
| 1. SIRT3 |
| 1. SKP2 |
| 1. SLCO1B3 |
| 1. SMAD4 |
| 1. SNAI2 |
| 1. SRC |
| 1. STAT3 |
| 1. STK11 |
| 1. TAGLN |
| 1. TGFB1 |
| 1. THBS1 |
| 1. TIMP1 |
| 1. TP53 |
| 1. TPM1 |
| 1. UCHL1 |
| 1. USP9X |
| 1. WISP3 |
| 1. WNT2 |
| 1. XIAP |
| 1. YWHAZ |
